# Shrub growth and plant diversity along an elevation gradient: Evidence of indirect effects of climate on alpine ecosystems

**DOI:** 10.1371/journal.pone.0196653

**Published:** 2018-04-26

**Authors:** Francesco Boscutti, Valentino Casolo, Paola Beraldo, Enrico Braidot, Marco Zancani, Christian Rixen

**Affiliations:** 1 Department of Agricultural, Food, Environmental and Animal Sciences, Plant Biology Unit, University of Udine, Udine, Italy; 2 WSL Institute for Forest, Snow and Landscape Research SLF, Unit Ecosystem Boundaries, Alpine Ecosystems, Davos, Switzerland; Ecole Pratique des Hautes Etudes, FRANCE

## Abstract

Enhanced shrub growth and expansion are widespread responses to climate warming in many arctic and alpine ecosystems. Warmer temperatures and shrub expansion could cause major changes in plant community structure, affecting both species composition and diversity. To improve our understanding of the ongoing changes in plant communities in alpine tundra, we studied interrelations among climate, shrub growth, shrub cover and plant diversity, using an elevation gradient as a proxy for climate conditions. Specifically, we analyzed growth of bilberry (*Vaccinium myrtillus* L.) and its associated plant communities along an elevation gradient of ca. 600 vertical meters in the eastern European Alps. We assessed the ramet age, ring width and shoot length of *V*. *myrtillus*, and the shrub cover and plant diversity of the community. At higher elevation, ramets of *V*. *myrtillus* were younger, with shorter shoots and narrower growth rings. Shoot length was positively related to shrub cover, but shrub cover did not show a direct relationship with elevation. A greater shrub cover had a negative effect on species richness, also affecting species composition (beta-diversity), but these variables were not influenced by elevation. Our findings suggest that changes in plant diversity are driven directly by shrub cover and only indirectly by climate, here represented by changes in elevation.

## Introduction

Plant species and communities in alpine and arctic environments are undergoing considerably changes (e.g. [1–4]). In both regions, a prominent piece of evidence of vegetation changes is shrub expansion [5–7] which can significantly alter ecosystem functioning and diversity [7–9]. Shrub expansion has most likely been driven by climate change [10,11], as shrub vegetation showed to be highly sensitive to changes in temperature [11–14].

Shrub annual growth and growth rings can be highly linked to climate and can represent year-to-year variation in temperature, where a general increase in shrub growth is expected as a major response to global warming [14,15]. In addition, the age of shrubs or of their ramets has been shown to reflect environmental conditions [16–18]. Other shrub traits, such as shoot length, leaf number, abundance and biomass, have commonly been found to be sensitive indicators of environmental change and ecosystem functioning [19–21]. Hence, studying such traits in combination with age distributions can improve our understanding on the ongoing changes in arctic and alpine ecosystems and give insights into population dynamics [5,22,23].

Changes in plant growth resulting from climate warming can cause considerable modifications in vegetation traits [24–26], which can in turn influence species composition, ecosystem functions and thus ecosystem services, such as regulation of nutrient cycles, gas exchanges or biomass stock [4,27]. One major effect of climate-related vegetation alteration is changes in biodiversity [1,28,29]. In particular, changes in the abundance and height of shrubs could lead to tangible shifts in both structure and species composition of a plant community [27,30].

Elevation gradients represent a powerful tool for investigating relationships between climate and vegetation [31]. In many studies, elevation has been used as a proxy for temperature because, on average, temperature drops by 5.5 K per vertical kilometer [31]. Changes in ecosystem properties along elevation gradients include plant diversity [32], productivity [33,34], species traits [35,36] and physiology [37]. Plant species richness is commonly thought to decrease with elevation [38–40]; however, several authors have shown the presence of a mid-elevation peak in species richness [41,42], yielding a humped relationship. In alpine areas, this peak has been found around the tree line [43], where both stress and competition are intermediate and habitat diversity is high. The relationship between elevation and species richness can also depend on the study scale [44], in particular in cases where important local factors (e.g. snowpack) vary drastically over short distances. However, plant communities along elevation gradients can also be shaped by a few key species such as dominant shrub species [27]. Hence, it is important to study traits of both individual key species and the entire plant community along an elevation gradient. Shrubs traits often considered in elevation studies are plant age and other dendrochronological [15,35], as well as morphological and physiological parameters [35,45]. Most existing studies have focused on the effects of elevation on a single species, showing its acclimation strategies along elevation, while little is known about elevation effects on multiple species, their community and the feedbacks between them (but see [46]).

In this study, we used a dendrochronological approach in subalpine ecotonal dwarf shrub communities to analyze the relationships between growth traits (i.e. age distribution, xylem rings width and shoot length) of *Vaccinium myrtillus* L., shrub cover and plant diversity along an elevation gradient. This approach allowed us to disentangle the possible effects of climate, using elevation as a proxy, and shrub abundance on plant diversity and vegetation composition in an alpine ecosystem. Specifically, we hypothesized that *V*. *myrtillus* ramets would be younger and smaller and overall shrub cover would be lower at higher elevation. We also expected *V*. *myrtillus* traits to be related to shrub cover, which, along with elevation, would affect species richness and composition (beta-diversity) of the whole community.

## Material and methods

### Study sites and plant communities

The study was carried out in two valleys (ca. 15 km apart) in the central part of the Carnic Alps (Friuli Venezia Giulia, Italy 12° 44’ 21” E 46° 38’ 01” N). Bedrock is mainly constituted of Paleozoic metamorphic siliceous sandstone and mudstone [47]. The area has a mean annual precipitation of 1400 mm and a mean annual air temperature of 3.8°C (climate station at 1750 m a.s.l.). The study was conducted in alpine dwarf shrub communities dominated by *V*. *myrtillus* and *Rhododendron ferrugineum* (*Rhododendro-Vaccinion*) [48–50]. The most frequent herbs at the sites were *Deschampsia flexuosa*, *Arnica montana*, *Carex sempervirens*, *Homogyne alpina*, and *Solidago virgaurea* subsp. *minuta*. The area was previously grazed by livestock, but this activity ceased at least 10 years before the surveys. Grazing by wild ungulates in the area is probable, but no direct evidence of grazing was found during the field surveys in the selected plots.

### Sampling design

Sampling was conducted using a nested design at the two study sites (i.e. valley), with 20 sampling plots each 25 m^2^ in area (5 x 5 m) selected along an elevation gradient spanning ca. 600 vertical meters (i.e. from 1690 to 2220 m a.s.l. in the first valley, and from 1560 to 2080 m a.s.l. in the second valley). Plots were selected using a vegetation map and a digital elevation model (DEM); using a GIS environment (ESRI—ArcGIS 10.0), the plots were randomly positioned within dwarf-shrub communities along elevation belts of ca. 30 m. The plots were selected based on the following criteria: (i) dwarf-shrub cover > 30% of the overall vegetation cover; (ii) aspect between east (90°) and south (180°); and (iii) slope between 20 and 30°. In one of the two valleys, three plots were discarded due to lack of suitable dwarf-shrub patches, resulting in a total of 37 plots. All samplings were completed in August 2014. All surveys were conducted under the supervision and permission of the “Provincia di Udine”, which was responsible for the area management and conservation. The study did not involve collection or damages to any endangered or protected species.

### Growth traits of *Vaccinium myrtillus* populations

Ten ramets of *Vaccinium myrtillus* were collected in each plot, clipping them at 5 cm below ground level. The longest new shoot (length increment from one year) of each ramet was measured in the field. All the collected stems were treated with glycerin-alcohol and embedded in paraffin. Cross-sections of 5 μm thickness were then cut from the basal 1.5 cm of each stem using a rotary microtome (Leica RM 2135). Sections were dried at 60°C for 2 hours. Afterward, they were immersed in Xylol I (2 min), Xylol II (2 min), ethanol solutions (100% I, 100% II, 95%, 80%, 50%; 1 min each) and distilled water (1 min). Sections were stained with toluidine blue to emphasize the growth ring structure. Images of cross sections were captured at x40–x200 magnification through a microscope (Leica DMLB—Leica Microsystems, Germany) with a digital camera (Leica ICC 50). Overall, 370 cross sections were analyzed. Images were used to visually count xylem rings and to measure ring widths in three radii per section, using the plug-in ObjectJ of the ImageJ software [51]. Ring counts and ring width were averaged for each ramet.

### Plant community

In each plot, the cover value of all plant species was visually estimated as a percentage of ground area, by using a 5% step scale. Dwarf shrub cover (%) was also recorded. Taxonomy and nomenclature were assigned following Flora Alpina [52]. Species richness was calculated for each plot as the number of occurring species.

### Data analysis

General linear mixed-effects models (LMMs) were used to test: (i) the effects of elevation on growth traits of *V*. *myrtillus* (i.e. ramet age, ring width and shoot length; three separate models); (ii) the effects of elevation and considered plant traits on shrub cover (one model); and (iii) the effects of elevation and shrub cover on alpha-diversity (i.e. species richness; one model). The ramet age pattern was preliminary evaluated as mean and standard deviation of the number of xylem rings, while in the final models we considered only mean age and standard deviation was added as supplementary result (S1 Fig). In all models, the valley was included as a random effect, evident outliers were discarded, and for all variables a quadratic term was preliminarily included to account for possible non-linear relationships. Despite the low number of levels, we decided to include valley as random factor to focus on the main effect of the tested hypothesis. Furthermore, we preliminary included the valley as fixed-effect to take into account of possible interactions between locality and tested variables. No significant interactions emerged, showing a consistence of results with the used LMMs. All statistical analyses were performed using the “nlme” package version 3.1–131 [53] in R [54]. For all models, we also preliminarily included aspect (cosine transformation–southness index) and slope of each plot as environmental covariates. No significant effect emerged, and thus covariates were dropped from the final models. In addition, the correlation between dwarf shrub cover and cover of the two most abundant shrub species (i.e. *V*. *myrtillus*: r = 0.5, p< 0.05, LMM, p< 0.05; and *Rhododendron ferrugineum*: r = 0.4, p< 0.05, LMM, p< 0.05) were analyzed. Moreover, all models were recalculated using *V*. *myrtillus* cover values instead of dwarf shrub cover; as the results were comparable, the two variables were considered interchangeable, using dwarf shrub cover in the presented models. We used general linear mixed-effects models (LMMs) to estimate model parameters, as model residuals did not violate any linear model assumption.

We tested the influence of elevation and shrub cover on plant beta-diversity, expressed as an index of floristic similarity decay. Beta-diversity was calculated using the Bray-Curtis dissimilarity index [55]. The analyses were performed by regression on distance matrices (MRM) [56], which estimate regressions between two matrices. The matrices contained distances or dissimilarities among all the pairwise combinations of the plots. The response matrix was the Bray-Curtis dissimilarity index, the explanatory matrices were the elevation difference (vertical distance in meters between each pair of plots) and shrub cover difference (difference in shrub cover between each pair of plots). MRMs were conducted separately for each response variable (i.e. beta-diversity vs. differences in shrub cover and beta-diversity vs. differences in elevation) and using a linear model with and without a quadratic term to account for possible non-linear relationships. Statistical significance was determined using permutation tests (n = 9999). The MRM analyses were conducted using R [54] with the “MRM” function in the “ecodist” package version 2.0.1 [57]. R^2^ values were used to determine the variance explained by the model.

Piecewise Structural Equation Models (piecewise SEM) were fitted to assess potential network causalities between the considered variables. Piecewise SEMs are an extension of traditional SEMs that allow one to encompass hierarchical data by piecing multiple LMMs into one causal framework [58]. From an overall model based on *a priori* knowledge of interactions with all hypothesized effects, we used Shipley’s test of direct separation to fit the model, which evaluates the probability that none of the paths missing from the hypothesized network contain useful information [59]. Model should be rejected if a chi-squared test of Fisher’s C statistic is below the significance level (p< 0.05), indicating that model is inconsistent with the data.

## Results

**Fig 1:**
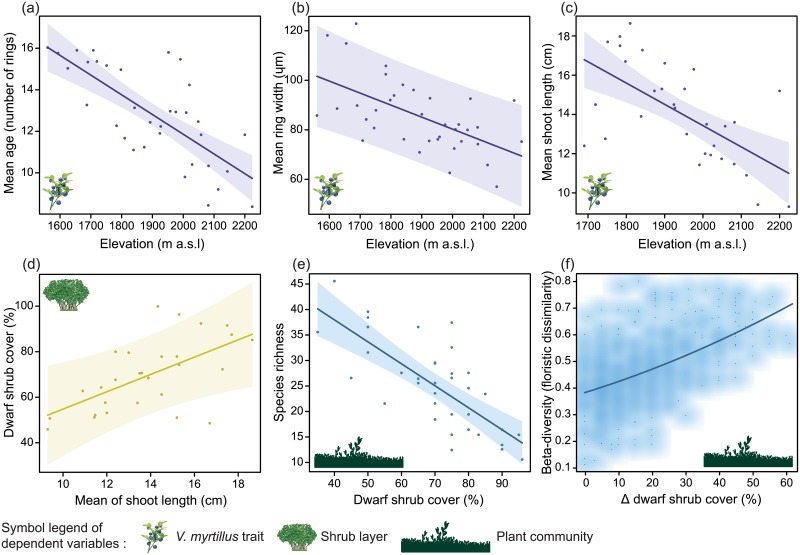
Effect of elevation on *Vaccinium myrtillus* traits (a, b, c), of *V*. *myrtillus* shoot length on shrub cover (d), and of shrub cover on species richness (e) and beta-diversity (f). Plots show the results of general linear mixed-effects models (a-e) and regression on distance matrices (i.e. floristic dissimilarity vs differences in shrub cover among all the pairwise combinations of the plots) (f). Confidence intervals (95%) are also shown (a-e). In the regression on distance matrices (f), the density of paired plots is represented by the intensity of the background color (smoothed scatter plot).

All the studied plant traits (i.e. ramet age, ring width, shoot length) showed significant negative relationships with elevation. At higher elevation, ramets of *V*. *myrtillus* were younger, with thinner growth rings and shorter shoots (Fig 1a–1c). *V*. *myrtillus* ramets had a mean age of 13.4 ± 3.2 years (max = 44 rings, min = 4 rings), which decreased ca. 1 year every 100 vertical meters. Ramet age was also more evenly distributed at high elevation (S1 Fig; r^2^ = 0.24, p = 0.001), where younger plants showed less variability in ring numbers. On average, rings were 84.8 ± 19.8 μm wide (max = 783.7 μm, min = 12.0 μm) and shoots were 14.5 ± 2.7 cm long (max = 25.0 cm, 7.0 cm), which decreased ca. 5 μm and 2 cm every 100 vertical meters, respectively.

Dwarf shrub cover showed a significant positive relationship with *V*. *myrtillus* shoot length (Fig 1d), whereas the effects of the other plant traits (i.e. ramet age and ring width) and elevation on shrub cover were not significant.

Species richness was lower in plots with higher shrub cover (Fig 1e), whereas no significant relationship with elevation was found. Species numbers ranged from 46 in plots with low shrub cover to 11 in plots where cover was high.

Beta-diversity (i.e. floristic dissimilarity) was most influenced by dissimilarities in dwarf shrub cover values (Δ dwarf shrub cover; r^2^ = 0.28, p = 0.001), showing an increase of floristic dissimilarity between pair of plots where differences of shrub cover for the same pair of plots were higher (Fig 1f). In contrast, the effect of elevation (Δ elevation) on beta-diversity was negligible (r^2^ = 0.02, p = 0.07).

**Fig 2:**
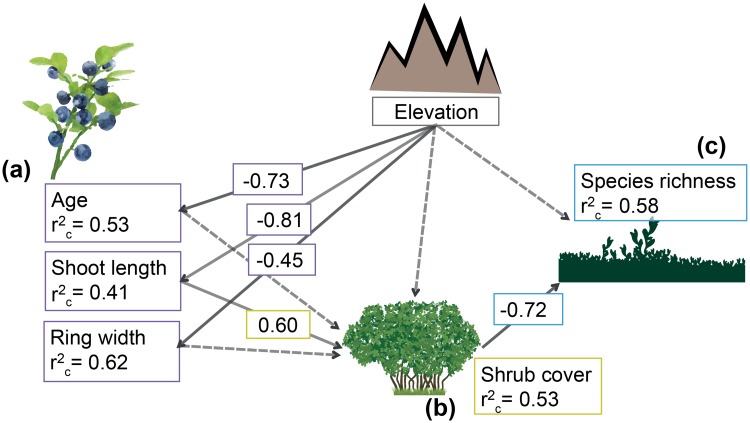
Structural equation model diagram showing the hypothesized relationships among elevation (gray box) and *Vaccinium myrtillus* growth traits (a, purple boxes), shrub cover (b, yellow boxes) and species richness (c, blue boxes). Solid lines indicate significant relationships (p<0.05), whereas dashed lines indicate tested relationships that were not statistically significant. Standardized effect size (i.e. scaled by mean and variance) of significant variables and conditional coefficient of determination (r^2^_c_) are also shown in the boxes.

Results of piecewise SEMs depicted possible causalities between the studied variables (Fisher’s C statistic = 11.46, p = 0.49). SEM analysis confirmed that elevation significantly affected all *V*. *myrtillus* growth traits but not shrub cover (Fig 2a). In turn, shrub cover was significantly related to *V*. *myrtillus* shoot length (Fig 2b). Species richness was significantly related to shrub cover but not to elevation (Fig 2c).

Complete outcomes of all the models tested in the Piecewise SEM are presented in the supplementary material S1 Table.

## Discussion

All the traits measured for *V*. *myrtillus* (ramet age, ring width, shoot length) were directly and negatively related to elevation. Elevation, however, did not affect shrub cover, which was positively related to *V*. *myrtillus* growth (i.e. shoot length). Dwarf shrub cover was the main driver of plant diversity, whereas elevation did not affect species richness or beta-diversity. These findings suggest that elevation directly influences *V*. *myrtillus* growth but not plant diversity. Hence, the growth of a key species, such as *V*. *myrtillus*, may overrule the effect of elevation on alpine shrub-dominated ecosystems.

As we hypothesized, all *V*. *myrtillus* growth traits decreased with increasing elevation, i.e. ramets were shorter and younger, and growth rings were thinner at higher elevation. These results support previous studies on *V*. *myrtillus* [21,35,45] and other vascular plants [15,60–62]. The observed trends in growth traits occur in parallel with changes in environmental conditions for plant growth with increasing elevation. In temperate seasonal zones, the atmospheric pressure and associated CO_2_ concentration, as well as temperature, length of the vegetation period and nutrient availability, usually decrease with increasing elevation, whereas annual precipitation, frequency of frost during the vegetation period and solar radiation tend to increase [61,63,64]. These factors affect plant growth by generating limiting conditions [60,63]. Among the plant traits, plant age distribution has been proved to be strongly affected by elevation. Hallinger et al. [62] proposed that a decrease in the estimated age of shrub individuals along the elevation gradient provides evidence that an upslope advance of the altitudinal shrub line is underway. An upward shift of some dwarf shrub species has already been shown using diachronic analyses of vegetation surveys in artic ecosystems (e.g. [29,64,65]). Although we collected ramets belowground, where effects of missing rings are less strong [66], ramet age might not actually reflect the age of plant individuals, as it is not know where below ground the oldest parts of this clonal plant is located. If the ramet age in our study reflects plant age, our findings could depict dynamics due to either (i) colonization by plants from lower to higher elevation or (ii) an increase in individual (and/or ramet) turnover due to environmental stress at higher elevation. Studies of other dwarf shrub communities have shown that, during colonization, pioneer stands have a peculiar age distribution pattern characterized by even-aged and very young individuals [67–69]. These dynamics are consistent with the ramet age distribution that we found, suggesting a plausible ongoing colonization of new stand of *V*. *myrtillus* at high elevation. At the same time, the lack of more detailed information concerning past grazing activities could not exclude an influence of land use abandonment on shrub encroachment, which could be more pronounced at high elevation, where grazing would have ceased first.

We also hypothesized that shrub cover would decrease at higher elevation like the other measured shrub traits, but this was not observed, as elevation and shrub cover were not directly related. Among the traits measured for *V*. *myrtillus*, only shoot length was related to overall shrub cover. We expected that elevation and decreasing temperature would directly affect shrub cover, reducing the cover of this life-form. On the other hand, previous studies have already demonstrated that climate effects can be remarkably blurred by other factors, such herbivores activity [70,71] or alterations in nutrients cycling [72,73]. Although no signs of grazing were found during our surveys, it is not possible to exclude the legacy effects of past land use on shrub abundance. Moreover, the effects of small-scale ecological features (e.g. soil conditions) can also have an important influence on shrub cover [70,74]. The finding that shrub cover was explained by changes in shoot growth of *V*. *myrtillus* points to the importance of understanding growth responses of key species.

In our study, the importance of growth of a key alpine species, *V*. *myrtillus*, was particularly relevant at the whole community scale: we found that dwarf shrub cover rather than elevation affected both species richness and beta-diversity by drastically reducing species number and shifting species composition. Changes in the dominance structure of the plant communities (i.e. where fewer species contribute a larger proportion of the total cover) can decrease the evenness of a community and trigger local extinctions of many species [27]. The lack of effect of elevation on species richness in our study may be due to factors like soil heterogeneity and grazing [75] or to a shift in the relative abundance of extant species [27,76]. The effect of shrub cover on beta-diversity was particularly apparent through an increase of floristic dissimilarity with increasing shrub cover heterogeneity (i.e. difference) between pairs of plots (Fig 1f). This finding highlights that a change in dwarf shrub cover had a significant effect on species turnover within the study community. This, coupled with the observed decrease of species richness, points to a homogenization of species composition, where few tall and competitive species could dominate and replace smaller light-tolerant species. Hence, dwarf shrubs can be considered as keystone species with considerable effects on other *taxa* and on ecosystem functions, such general matter and energy fluxes [77]. Changes in the abundance and height of shrubs could lead to considerable shifts in both the structure and species composition of a plant community [27,30,78]. Our findings support that shrub encroachment may cause a decline in biodiversity across a wide variety of tundra ecosystems, at least over the short term [27].

## Conclusions

In this study of alpine vegetation, higher shrub cover had negative effects on species richness drastically affecting also plant community (beta- diversity), which were not influenced by elevation. These findings indicate that changes in cover of dwarf shrubs can cause species replacement in plant communities, leading to a significant decrease in species richness. This process seems to be indirectly mediated by an effect of elevation on the growth of key species. In fact, *V*. *myrtillus* shoot growth enhanced shrub cover, which in turn decreased plant species richness. Our results demonstrate the importance of studying a wide range of variables, from key species traits to community structure, to interpret changes in ecosystems. Our findings, hence, suggest that changes in plant diversity are directly driven by shrub cover and only indirectly by climate changes, here represented by the elevation gradient.

## Supporting information

S1 Table Complete outcomes of all the models tested in the piecewise SEM. (DOCX)

S1 Fig Linear relationship between standard deviation of the ramet age and the elevation. Results were calculated with general linear mixed-effects models. Confidence intervals (95%) are also shown.
(EPS)

S1 Dataset Data and species matrix. (XLSX)

S1 R Script Complete R Script. (R)
