# Supplementary material for: Shrub growth and plant diversity along an elevation gradient: Evidence of indirect effects of climate on alpine ecosystems
Source: PLoS One. 2018 Apr 26;13(4):e0196653. doi: 10.1371/journal.pone.0196653 (PMC5919657; doi:10.1371/journal.pone.0196653)
Supplement: S1 Table — (DOCX) [file pone.0196653.s001.docx]

Boscutti et al., Shrub growth and plant diversity along elevation - Supporting information

**S3 Table. Complete outcomes of all the models tested in the Piecewise SEM.** Model formula, Conditional R^2^, predictor names and their standardize effect size (estimates), errors (std.error) and p-values (p.value) are shown.

| Model | Conditional R^2^ | predictor | estimate | std.error | p.value |
| --- | --- | --- | --- | --- | --- |
| Ramet age ~ Elevation | 0.53 | Elevation | -0.74 | 0.12 | <0.001 |
| Ring width ~ Elevation | 0.62 | Elevation | -0.46 | 0.12 | <0.001 |
| Shoot length ~ Elevation | 0.41 | Elevation | -0.82 | 0.19 | <0.001 |
| Dwarf shrub cover ~ Elevation + Ramet age + Ring width + Shoot length | 0.54 | Shoot length | 0.60 | 0.25 | 0.024 |
|  |  | Ring width | -0.62 | 0.36 | 0.1 |
|  |  | Ramet age | 0.31 | 0.25 | 0.224 |
|  |  | Elevation | 0.35 | 0.34 | 0.32 |
| Species richness ~ Elevation + Dwarf shrub cover | 0.59 | Dwarf shrub cover | -0.72 | 0.12 | <0.001 |
|  |  | Altitude | 0.06 | 0.12 | 0.627 |
